# Increasing landslide susceptibility and intensity under climate change for Aotearoa New Zealand

**DOI:** 10.1038/s41598-026-46684-7

**Published:** 2026-04-07

**Authors:** Livio Dreyer, Thomas R Robinson, Marwan Katurji, Kerry Leith, James H Williams

**Affiliations:** 1 University of Canterbury, Christchurch, New Zealand; 2 Earth Sciences New Zealand, Lower Hutt, New Zealand

**Keywords:** Climate sciences, Environmental sciences, Natural hazards

## Abstract

Extreme weather events like tropical cyclones can trigger large numbers of landslides across vast areas due to extreme rainfall. Cyclone Gabrielle in February 2023 triggered ~ 800,000 landslides across the North Island of New Zealand and is one of the most extreme landslide-triggering storms recorded to date. While climate change is expected to increase the intensity of future tropical cyclones, whether this translates into increased landslide numbers from extratropical cyclonic movement towards the New Zealand landmass remains contentious. Here we show that under a + 2 °C warming scenario, a Cyclone Gabrielle-like storm could trigger up to ~ 90,000 more landslides than the 2023 event, while the area experiencing extreme landslide densities might increase by up to 34%. Our findings suggest significant increases in landslide hazards due to climate change, providing an essential evidence base for land-use planning and targeted mitigation efforts in locations where landslide hazards are projected to intensify.

**Supplementary Information:**

The online version contains supplementary material available at 10.1038/s41598-026-46684-7.

## Introduction

Shallow landslides are widespread and destructive geohazards globally, causing numerous human casualties, infrastructure damage, and economic losses^1–3^. Current approaches to landslide hazard assessment at regional scales often fail to adequately integrate the essential spatial, temporal, and intensity dimensions required for comprehensive hazard evaluation. Traditional methods either rely on static environmental factors while neglecting rainfall dynamics for binary landslide susceptibility modelling^4^ or focus on empirical rainfall thresholds that oversimplify spatial heterogeneity and neglect hydrological preconditioning^5–7^. Recent advances have begun integrating dynamic rainfall data with spatial susceptibility models^8–10^ and addressing landslide intensity predictions rather than just binary classification^11–13^. However, the complex relationship between rainfall variability and landslide susceptibility and intensity remains poorly quantified, partly due to significant challenges in acquiring accurate, spatially distributed rainfall estimates across affected regions^7,14^. Understanding how rainfall modulates landslide susceptibility and intensity is crucial for forecasting future hazards, particularly in the context of climate change.

**Fig. 1:**
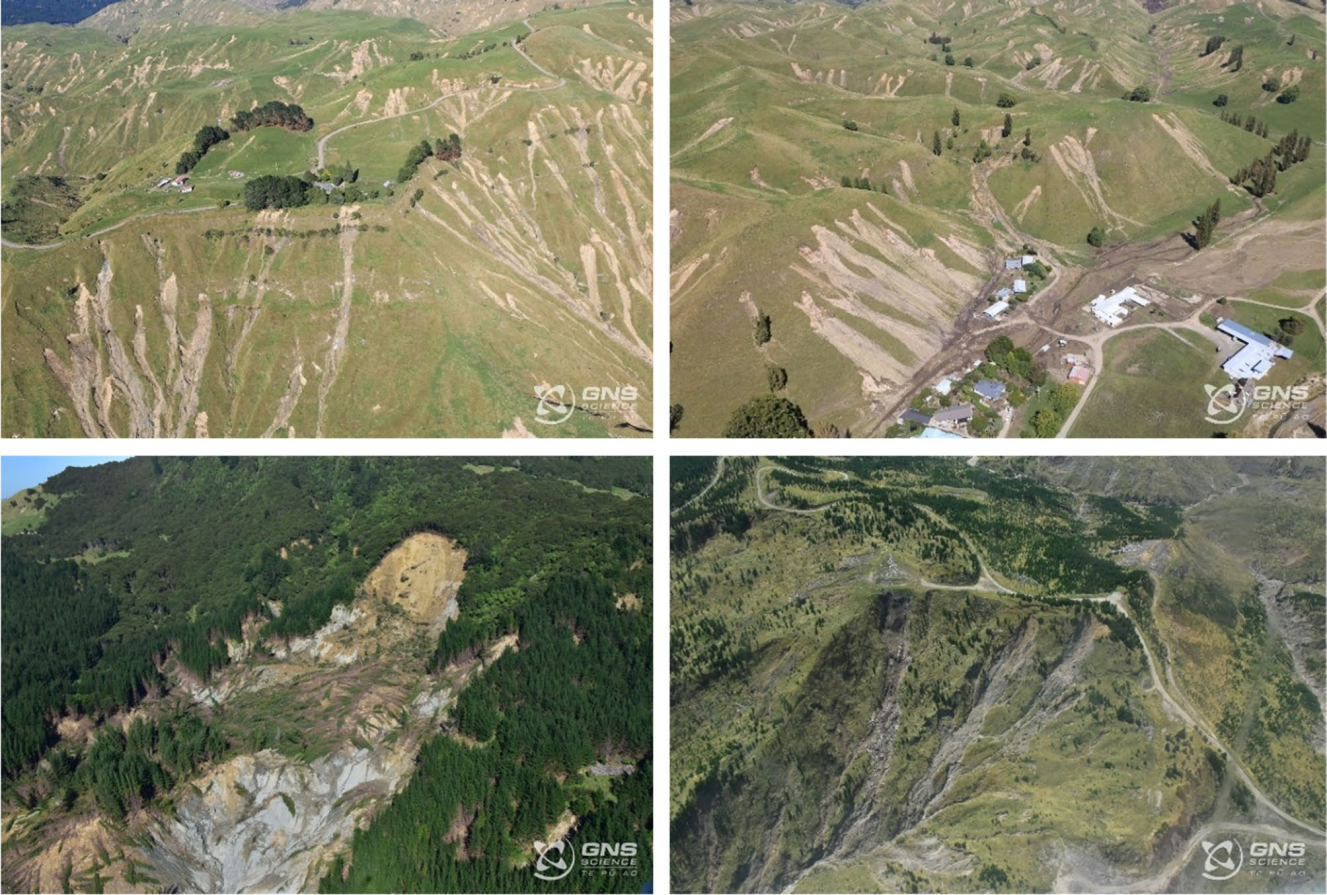
Aerial photographs after Cyclone Gabrielle in the Hawke’s Bay and Tairāwhiti regions. ^38^. *Source*: GNS (2025).

**Fig. 2:**
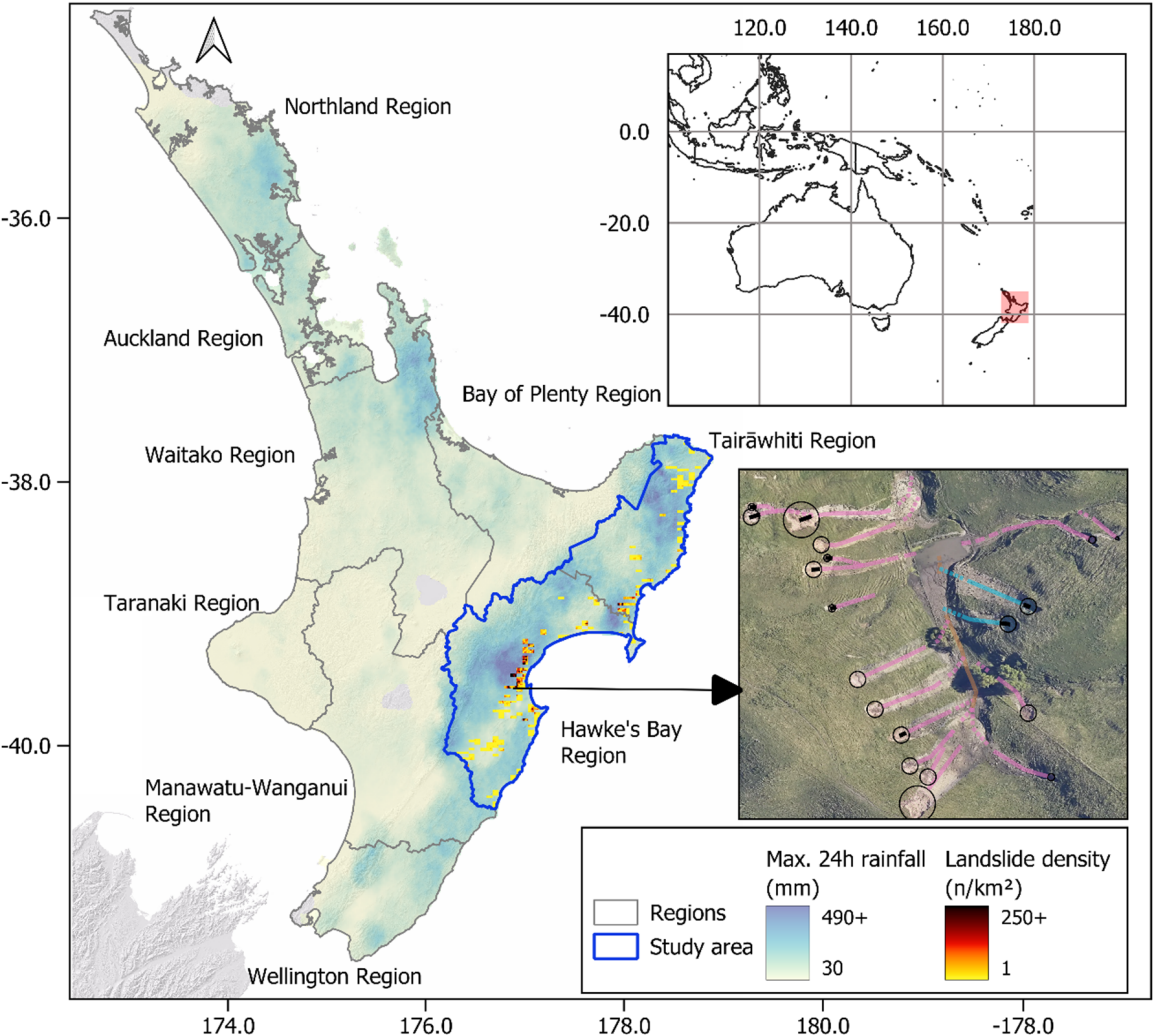
Overview of study area (Hawke’s Bay and Tairāwhiti region, blue outline) and mapped landslide grids with corresponding landslide densities used for model training and validation. Inset shows an example of mapped landslides using the mapping approach of Leith et al.^15^. Blue lines = Slides, Pink Lines = Flows.

Aotearoa New Zealand is especially prone to shallow landslides due to its steep topography, high seismicity, and frequent severe weather events. Between February 12–16, 2023, Cyclone Gabrielle triggered one of the largest landslide events on record in Aotearoa New Zealand, producing an estimated 800,000 landslides across the North Island^15^ (Figs. 1 and 2). The Hawke’s Bay and Tairāwhiti regions experienced exceptionally high rainfall with local 24-h rainfall sums exceeding 490 mm (Fig. 1). This resulted in widespread landslides with densities exceeding 300 landslides per km^2^ (Fig. 2), some of the highest landslide densities recorded globally for a rainfall-triggered landslide event^16^. The region had already experienced elevated rainfall, including from Cyclone Hale just four weeks prior^17^. In conjunction with flooding, Cyclone Gabrielle caused infrastructure damage, loss of life, and economic losses surpassing NZ$1.83 billion in insurance claims^18^.

Climate change projections for Aotearoa New Zealand indicate an increase in the frequency and intensity of extreme rainfall events^19–21^. These changes are expected to amplify landslide hazards across susceptible landscapes, potentially increasing both the spatial extent of vulnerable areas^22,23^ and landslide intensities^9,24^. Event-scale attribution^25^ and high-resolution Weather Research and Forecasting (WRF) experiments^26^ indicate that the rainfall associated with Cyclone Gabrielle has likely already been intensified by anthropogenic warming. Although studies investigating changes in rainfall triggered landslides under climate change have recently increased, the uncertainties are still substantial. Studies often project different, and sometimes opposite changes in landslide occurrence, mainly due to different geological and climate conditions, the assumptions included in the study design, or accounting for climate change projection uncertainty^27^. Moreover, most studies focus on long-term changes in future landslide susceptibility rather than individual event-scale extremes. Rainfall projections from downscaled global circulation models (GCMs) are frequently used^28–30^ which may exhibit significant biases in their representation of extreme rainfall events^31–33^. It is therefore crucial to quantify plausible changes in extreme events characteristics and their direct impact on landslide occurrence and intensity.

To address this, we take an event-based storyline approach^34,35^, a method increasingly used to represent climate change uncertainty and assess extreme event related impacts^27,36,37^ by exploring a physically plausible future. We seek to understand how extreme storm rainfall influences landslide susceptibility and intensity, and the associated landslide hazard if a Cyclone Gabrielle-type event would occur in a world that is 2 °C warmer than pre-industrial levels. Within this framework we aim to (1) quantify the modulation of landslide initiation and spatial patterns by dynamic rainfall characteristics; (2) establish relationships between rainfall intensity and landslide size as a function of planimetric area; and (3) using a storyline approach, project landslide susceptibility and intensities under a + 2 °C warming scenario.

We focus our study on the Hawke’s Bay and Tairāwhiti regions on Aotearoa New Zealand’s North Island (Fig. 2). We leverage the extensive Cyclone Gabrielle landslide inventory and high-resolution rainfall data to derive robust statistical relationships between rainfall metrics and landslide susceptibility and intensity at the regional scale using generalised additive models (GAMs). Through a storyline approach, the developed models enable the prediction of landslide susceptibility and intensities under a future storm event, using Cyclone Gabrielle-like storm data as a contemporary analogue for future extreme events. As part of our storyline approach, we also explore the spatial distribution of newly susceptible areas in relation to present susceptible areas, providing critical insights for hazard mitigation in a warming climate.

## Methods

### Data

Following Cyclone Gabrielle, Leith et al.^15^ created a landslide inventory comprising over 145,000 mapped landslides across Hawke’s Bay, Tairāwhiti, and Auckland regions, representing one of the largest event-specific landslide catalogues globally. Mapping used a systematic grid cell based approach, mapping a total of 663 12 × 5 km grid cells across the North Island. We selected a subset of 182 quality-controlled grid cells within our study area containing 43,336 landslides (flow and slide types only) for our analysis (Fig. 2).

For rainfall data, we used high-resolution (1 km) Quantitative Precipitation Estimates (QPE) provided by MetService^17^ which blend rain gauge observations, weather radar, and satellite/ Numerical Weather Prediction (NWP) data. We selected maximum 24-h rainfall as the primary triggering variable and calculated antecedent rainfall over 7-, 15-, 25-, and 35-day windows. We also considered long-term rainfall variables including annual rainfall^39^, heavy rainfall frequency^40^, and heavy rainfall^40^ (99th percentile).

Drawing on established relationships between static factors and landslide susceptibility in Aotearoa New Zealand^9,41–43^ we selected key geomorphic variables—slope, aspect, curvature (derived from 1 m resolution LiDAR data), land cover, and lithology—for the static model component (1). We derived rock types from the New Zealand Land Resource Inventory^44^ and landcover from the LUCAS NZ Land Use Map v005^45^. Since preparation of the land use dataset in 2020/2021 significant areas of plantation forest have been harvested, so we update these areas with the LUCAS NZ Forest Clearing dataset^46^ to more accurately reflect the land use at the time of Cyclone Gabrielle.Table 1 gives an overview of the used data and sources in this study.

**Table 1:** Overview of used data and sources in this study.

Data	Sources
Landslide inventory	Leith et al.^15^
Event rainfall (QPE)	MetService^17^
Pre-event rainfall (QPE)	MetService^17^
Heavy rainfall frequency	Gibson et al.^40^
Rock type	Newsome et al.^44^
Landcover	Ministry for the environment^45,46^
Digital elevation model (DEM)	Land Information New Zealand^47,48^
Gabrielle Design Storms (WRF)	Stone et al.^26^

We assess landslide susceptibility and intensity under a future Cyclone Gabrielle-like event using Weather Research and Forecasting (WRF) model simulations from Stone et al.^26^. We use an actual simulation, driven by the NCEP Global Forecast System (GFS) boundary conditions and + 2.0 °C design storms representing a future climate ~ 2.0 °C above pre-industrial levels (~ 1.0 °C warmer than present). Simulations from Stone et al.^26^ were conducted using a two-way nested WRF configuration with 24 km (outer domain) and 8 km (inner domain) resolutions. The + 2.0 °C design storms outcomes from WRF resulted from modifying the actual boundary conditions by uniformly applying a Coupled Model Intercomparison Project Phase 5 (CMIP5)-derived regional anthropogenic warming signal (~ 2.0 °C), isolating thermodynamic impacts while preserving storm dynamics. The scenarios we used were based on Stone et al.^26^: a deterministic simulation not subjected to stochastic perturbation, and an ensemble mean of 20 members, which were stochastically perturbated to account for sub grid-scale process uncertainty that better capture a range of possible simulation outcomes.

### Statistical landslide modelling approach

To capture different geomorphological representations, we used two mapping units: 5 m grid cells and geomorphologically relevant slope units. A total of 437,039 slope units (mean (μ) = 0.051 km^2^, standard deviation (σ) = 0.039 km^2^) were delineated across the AOI using the r.slopeunits algorithm^49^. To model landslide susceptibility and intensity during Cyclone Gabrielle we developed three Generalised Additive Models (GAMs):

1. A binary susceptibility model using grid pixels (5 m) to understand and isolate the influence of topography at high resolution
2. A binary susceptibility model at the slope unit level that better captures geomorphological contributions and improves accuracy of the landslide inventory position.
3. A log-gaussian size model that predicts the total planimetric landslide source area per slope unit. Throughout the manuscript we refer the slope unit size model and its related outputs as the landslide intensity model and landslide intensity respectively.

We employed GAMs as our primary statistical approach due to their ability to capture non-linear relationships between predictors and their outcomes without requiring a priori knowledge of the functional form of these relationships^50,51^. GAMs extend generalised linear models by allowing the linear predictor to include smooth functions of predictor variables, making them particularly well-suited for modelling complex environmental relationships. They have been effectively used in a wide range of environments for both landslide susceptibility^52,53^ and landslide intensity modelling^13,54,57^. For susceptibility modelling, a binomial GAM with a logit link:

1 $$g\left( \mu \right) = {\mathrm{log}}\left( {\frac{\mu }{1 - \mu }} \right)$$

was used to predict landslide occurrence (binary outcome P) as:

2 $$\log it(P) \, = \, \alpha \, + \mathop \sum \limits_{i = 1}^{k} f_{i} \left( {x_{i} } \right) + \mathop \sum \limits_{i = 1}^{P} a_{j} x_{j}$$

where $g\left( \mu \right)$ is the logit link, α is the global intercept, $f_{i} \left( {x_{i} } \right)$ the smooth function for nonlinear covariates $x_{i}$, and $a_{j} x_{j}$ denotes the regression coefficient for linear covariates. The target variable P is assumed to be distributed as Bernoulli distribution.

For landslide size modelling, we assumed a log-Gaussian distribution for the total planimetric landslide area (A) within slope units^12^. The log-Gaussian GAM structure is given by:

3 $$\begin{gathered} \log \left( A \right)\sim N \left( {\mu , \sigma^{2} } \right), \hfill \\ g\left( \mu \right) = \beta + \mathop \sum \limits_{i = 1}^{k} f_{i} \left( {x_{i} } \right) + \mathop \sum \limits_{i = 1}^{P} a_{j} x_{j} \hfill \\ \end{gathered}$$

where $\mu , \sigma^{2}$ are the mean and variance of the gaussian distribution and $\beta$ the global intercept, $f_{i} \left( {x_{i} } \right)$ the smooth function for nonlinear covariates $x_{i}$ and $a_{j} x_{j}$ the regression coefficient for linear covariates similar to the binomial GAM. Models were implemented using the mgcv package^51^ (v1.9.3) in the R programming language. We use fivefold random and k-means spatial cross-validation (10 repetitions) to assess the model performance. Data preparation and variable selection procedures are detailed in the Supplementary Information.

### Storylines: + 2 °C design storms and landslide response

In a future world, where Cyclone Gabrielle’s intensity is expected to increase due to warmer and moister tropospheric conditions, we are expecting an increase in landslide susceptibility and intensity. To evaluate the impact of increased rainfall on the landslide models, we applied rainfall inputs from the actual Cyclone Gabrielle WRF and + 2 °C warming design storms to calibrated susceptibility and intensity slope unit models, using uniform coarse-resolution (12 km) rainfall data.

Changes of regional-scale predictions of total planimetric landslide source area per slope unit are challenging to interpret due to varying slope unit sizes; thus, we converted these values to landslide counts. Following Di Napoli et al.^11^ we tested various statistical distributions suitable for heavy-tailed data distributions and selected a log-Gaussian distribution to estimate the mean landslide size. Dividing the total predicted area by the mean landslide source area yielded landslide counts per slope unit. For visualisation, we standardised the landslide counts per slope unit to landslide density (number/km^2^), which is a widely used metric to express the spatial concentration of slope failures.

Finally, we combined susceptibility and intensity outputs from the grid- and slope unit-based models to identify the most probable landslide area under the deterministic + 2.0 °C storyline storm. We achieved this by populating the highest susceptibility pixels until we reached the predicted planimetric area per slope unit, which essentially serves as a lower cutoff threshold.

## Results

### Statistical landslide modelling: rainfall-landslide relationships

**Fig. 3:**
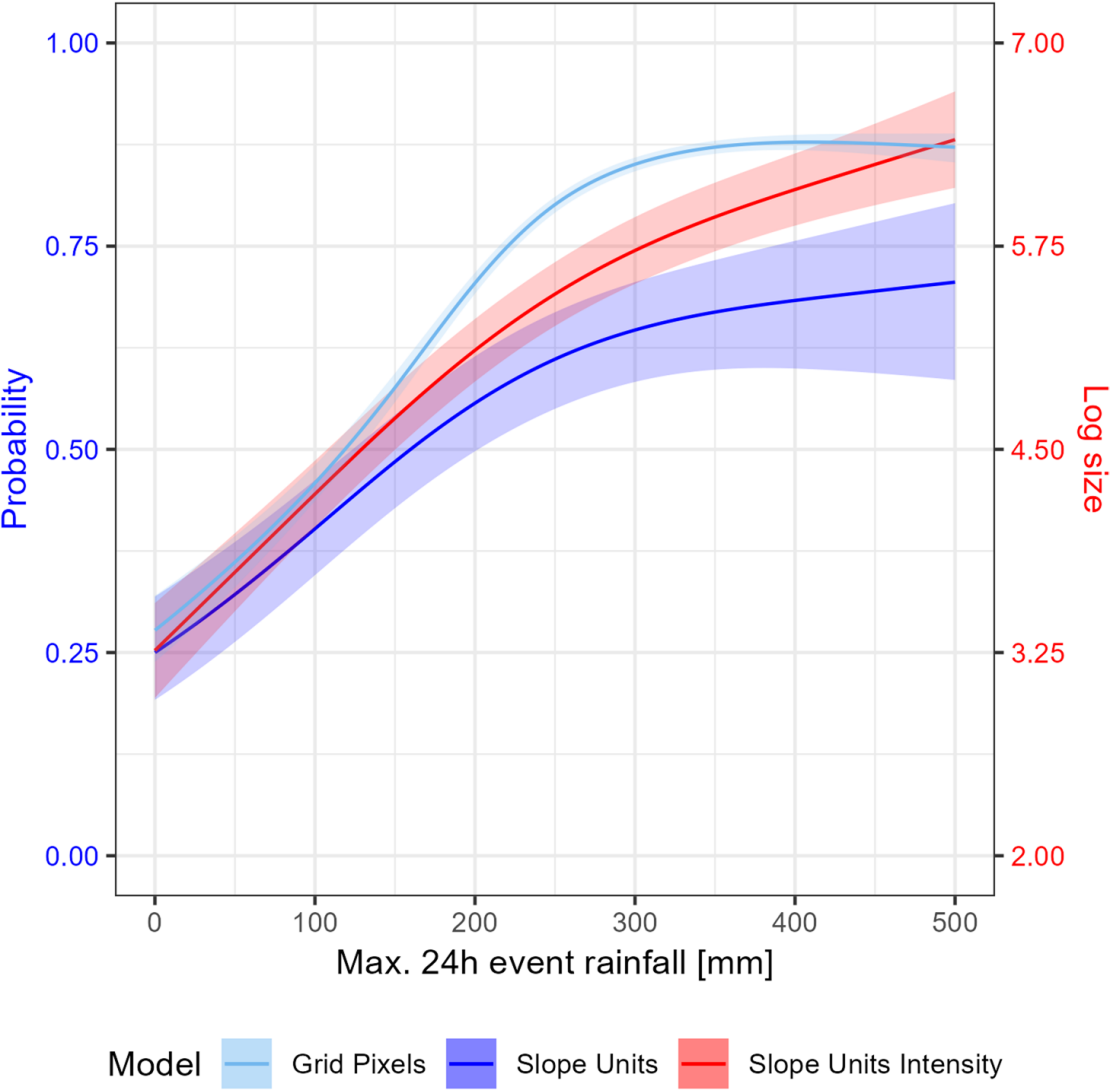
Partial dependence plot for the Maximum 24 h rainfall variable showing the model responses, while holding the remaining variables at their average. For the binary gridpixel and slopeunit models the model outcome is expressed at the response scale (probability), for the log gaussian slopeunit model the model results is represented as the planimetric landslide source area per slope unit on the log scale.

Our models reveal a threshold-dependent relationship between rainfall and landslide response. Partial dependence plots show that landslide probability increased linearly with maximum 24-h rainfall intensity up to approximately 300 mm, beyond which probabilities and intensities (total planimetric source area per slope unit) plateaued (Fig. 3). The influence of rainfall was strongly modulated by topography. Notably, we observe strong variations in the model outcome for the different variables for the same triggering rainfall amount. For example, 2D partial dependence plots (Figs. S4, S5) show that combinations of extreme rainfall (> 300 mm/24 h) and steep slopes (> 20°) yielded the highest landslide probabilities (> 0.9) in the binary slope-unit model, while flat areas exhibited near-zero probabilities. Other topographic variables, such as aspect, exerted much lower influence compared to slope steepness. Antecedent metrics (35-day cumulative rainfall) and heavy-rainfall frequency produced more complex, non-monotonic or weaker relationships (see Supplementary Fig.S3). Analysis of variable importance (Supplementary Fig. S2) revealed that topographic factors, particularly slope, had the largest influence on susceptibility, while the combination of maximum 24-h rainfall and slope angle jointly had the highest importance on the concentration of landslides in each area. The final selection of variables for each GAM model is found in Supplementary Table S1.

### Model validation

**Fig. 4:**
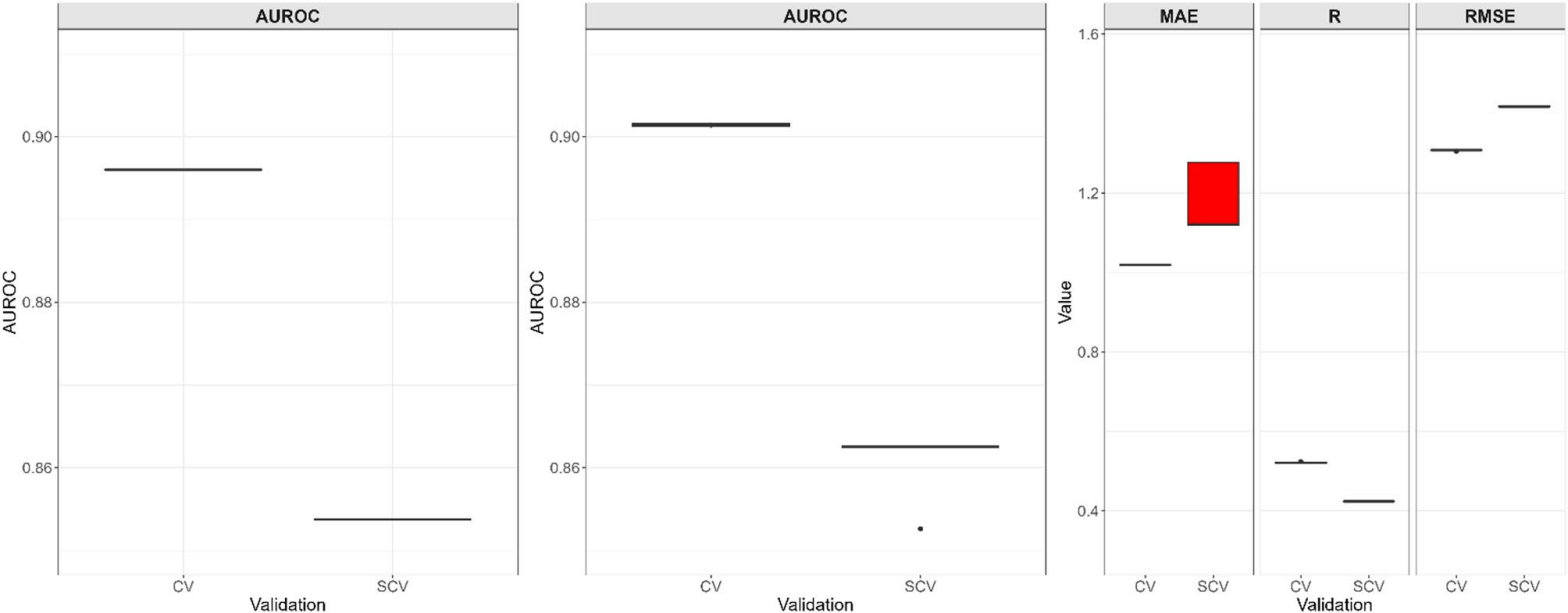
Validation metrics for random cross-validation (CV) and k-fold spatial cross-validation (SCV) for (**a**) binary gridpixel, (**b**) binary slopeunit and (**c**) log-gaussian model.

Model validation showed strong performance for susceptibility models (AUROC: ~ 0.9 for random CV; 0.84–0.85 for spatial CV), with expected declines under spatial cross-validation due to the breakdown of autocorrelation effects^55^. Compared to random CV, spatial CV gives a more realistic model performance assessment of how well the model will perform when applied outside of the training area. The landslide size model achieved moderate accuracy (CV: Mean Absolute Error (MAE) = 0.99, Pearson correlation coefficient (*R)* = 0.45, Root Mean Squared Error (RMSE) = 1.25), with slight reductions in spatial CV (Fig. 4).

### Spatial pattern of landslide susceptibility and area prediction for cyclone Gabrielle

**Fig. 5:**
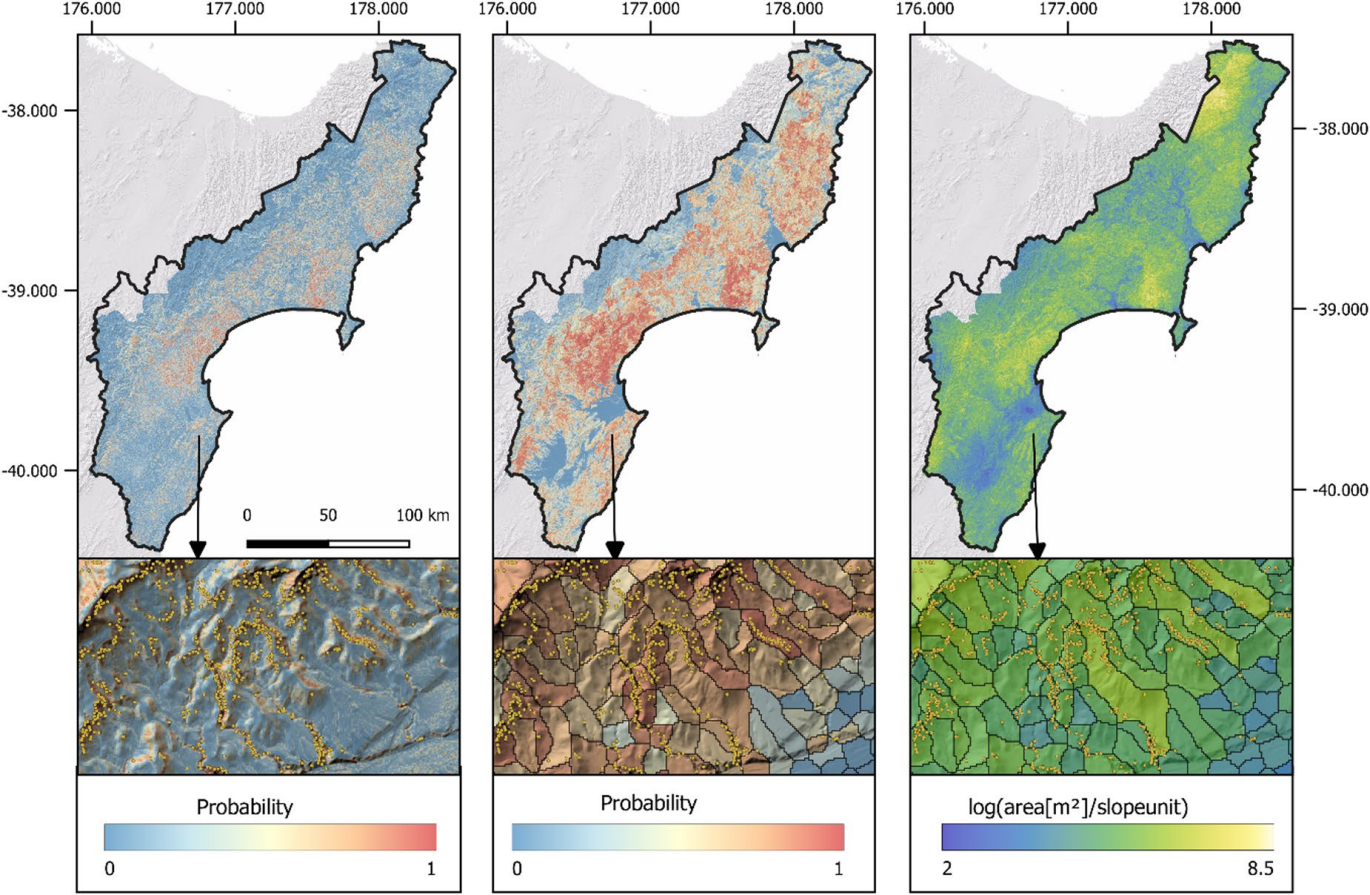
Landslide probability maps calculated from Cyclone Gabrielle rainfall using a grid pixel (left) and a slopeunit (middle) model. Right map shows the predicted total planimetric landslide area per slope unit on the log scale for the slopeunit intensity model. Yellow dots on inset maps show locations of mapped landslides from Leith et al.^15^.

We use the fitted GAM models to produce maps of predicted landslide susceptibility and intensity covering the entirety of Hawke’s Bay and Tairāwhiti Regions (Fig. 5). Spatial patterns reflect combined influences of extreme rainfall during Cyclone Gabrielle and terrain characteristics. Highest susceptibility values for both the grid pixel and slope unit models were predicted in eastern regions that were exposed to peak rainfall intensities and steep areas of northern Tairāwhiti and southwestern Hawke’s Bay. Despite comparable rainfall to central Hawke’s Bay, Tairāwhiti exhibited lower predicted landslide susceptibility despite its steeper terrain.

Across the training area, our model predicts a total landslide area of 4.7 km^2^ from ~ 42,000 landslides compared to a manually mapped area of 4.9 km^2^ from ~ 43,000 landslides (Table 2). Expanded to the entire study region, the model predicts total landslide area of 73.4 km^2^ from ~ 680,000 landslides. With previous estimates suggesting ~ 800,000 landslides were triggered by Cyclone Gabrielle across the entire North Island^56^, this highlights the Hawke’s Bay and Tairāwhiti Regions as being the most badly impacted, with ~ 85% of all triggered landslides occurring here.

**Table 2:** Change in total planimetric source area and total landslide numbers for deterministic and ensemble mean + 2.0 °C WRF forecast compared to actual Cyclone Gabrielle WRF forecast.

Sources	Total source planimetric area [km^2^]	Total landslide number
Mapped area		
Inventory	4.9	43,263
Deterministic actual WRF forecast	4.7	42,015
Study area		
Deterministic actual WRF forecast	73.4	684,504
Ensemble mean + 2.0 °C WRF forecast	78.2	732,388
Deterministic + 2.0 °C WRF forecast	82.9	777,564

### Landslide storylines for + 2.0 °C design storms

Storylines of Cyclone Gabrielle under a + 2 °C warming from Stone et al.^26^ project significant rainfall redistribution, with thermodynamic enhancement intensifying rainfall in the storm core while reducing it in peripheral regions..

**Fig. 6:**
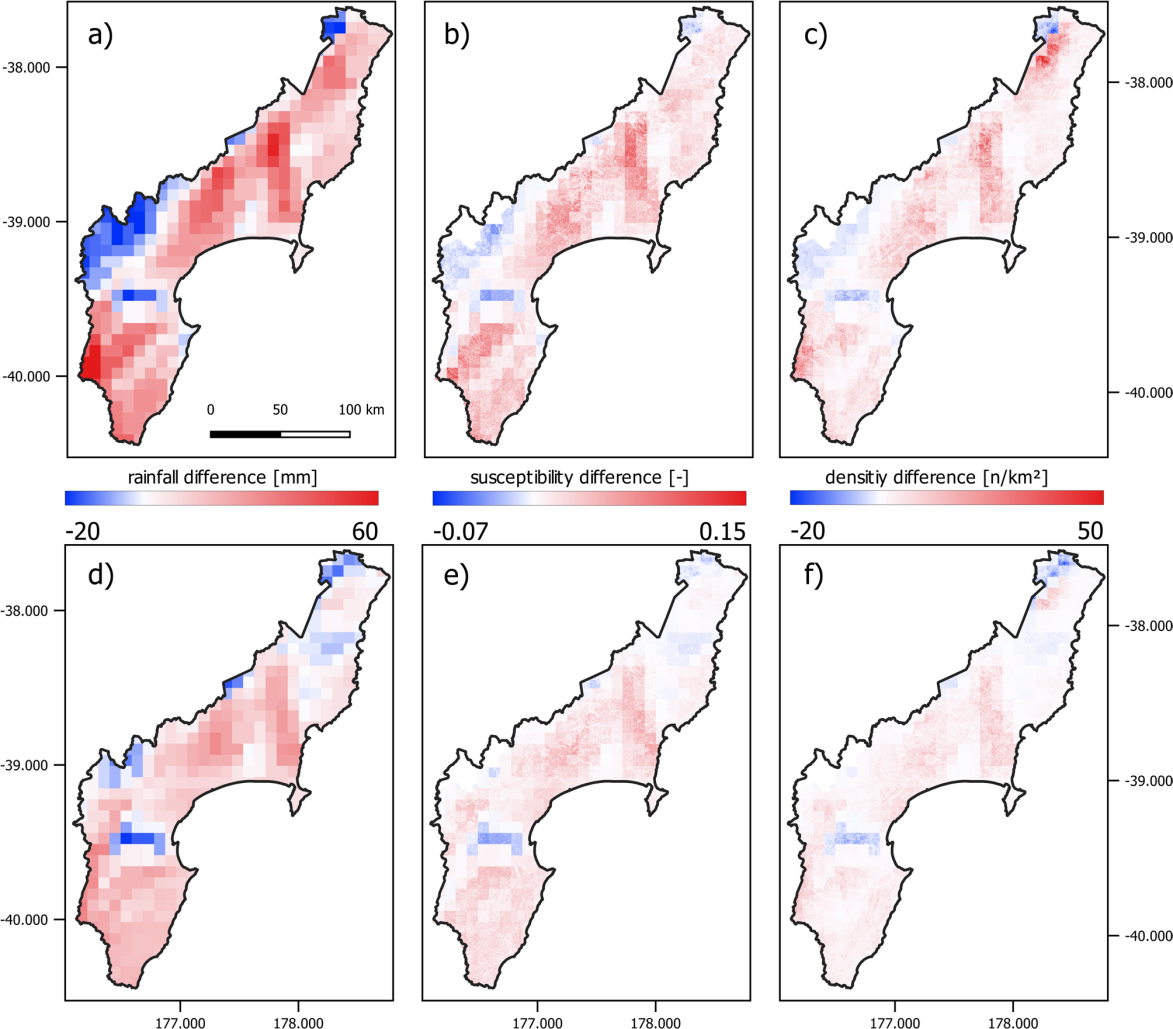
Maps of absolute differences in rainfall (**a**) and (**d**), susceptibility (**b**) and (**e**) and density (**c**) and (**f**) between actual WRF forecast and + 2.0 °C design storms. Top rows (**a**–**c**) show differences for the deterministic + 2.0 °C storm and the bottom row (**d**–**f**) for the perturbated + 2.0 °C ensemble mean. Top row and bottom row share the same legend for each variable.

Figure 6a–c shows the corresponding spatial pattern of the differences in modelled rainfall accumulation, as well as resulting landslide susceptibility and densities between the + 2 °C design storms and the actual WRF forecast for Cyclone Gabrielle. Table 2 provides quantitative estimates of some key summary statistics. The total number of landslides across the entire study area under a + 2.0 °C design storm storylines is predicted to increase by 7% (~ 732,000) for the ensemble mean storyline and 14% (~ 777,000) for the deterministic storyline, with forecast landslide areas of ~ 80 km^2^.

Table 3 summarises the change in areal extent across the study area exceeding extreme thresholds—defined by the 95th percentile—for rainfall, susceptibility, and landslide density for the deterministic and ensemble mean + 2.0 °C WRF forecast design storms compared to the actual forecast of Cyclone Gabrielle. The deterministic + 2.0 °C WRF forecast increased the area exceeding the 95th percentile susceptibility threshold from 5.0 to 6.3%, and the 95th percentile density threshold (> 86 landslides/km^2^) from 5.0 to 6.7%.

**Table 3:** Change in areas across the study area exceeding extreme (corresponding to the 95th percentile) rainfall, susceptibility and landslide densities for deterministic and ensemble mean + 2.0 °C WRF forecast compared to actual Cyclone Gabrielle WRF forecast (relative increase in brackets).

	Area exceeding 95% percentile		
	Rainfall [%]	Susceptibility [%]	Density [%]
Deterministic actual WRF forecast	4.5	5	5
Ensemble mean + 2.0 °C WRF forecast	5.6 [25.8%]	5.7 [14%]	5.7 [14%]
Deterministic + 2.0 °C WRF forecast	5.9 [31.8%]	6.3 [25.6%]	6.7 [34.6%]

**Fig. 7:**
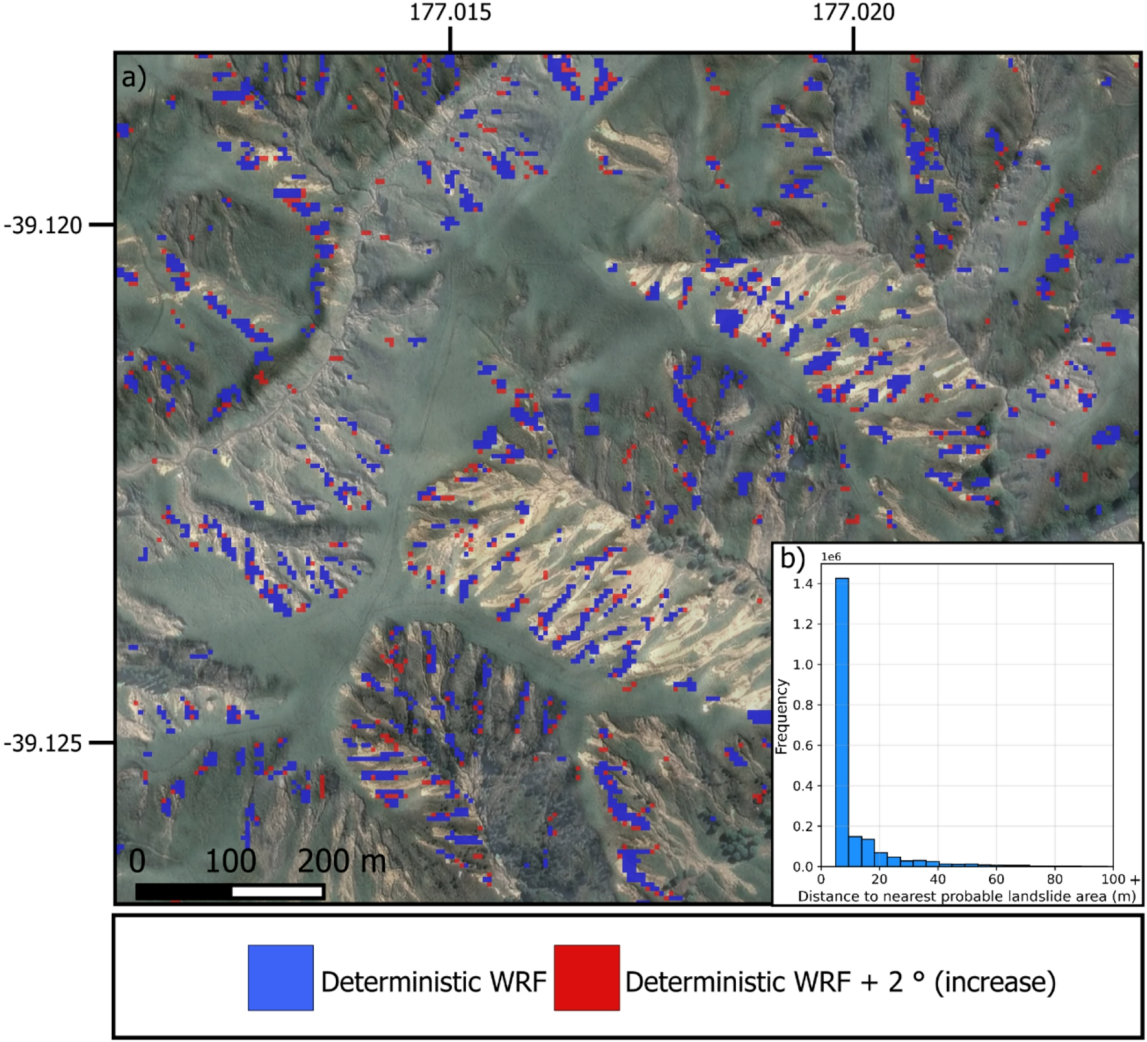
Visualisation of changes of probable landslide areas combining the predicted planimetric area with the highest susceptibility values for deterministic + 2 °C scenario (corresponds to an increase of 26 mm rainfall in this location). Blue shows the most probable pixels based on the actual WRF forecast and red the increase of new pixels under the deterministic + 2 °C scenario.

Our analysis shows that most probable new landslide areas projected under the deterministic warming storyline tend to cluster within or near existing high-susceptibility areas. Fig. 7 gives an example illustrating these changes. To quantify this clustering effect, we calculated the distances between each new probable landslide area projected under the deterministic + 2 °C storyline and the nearest probable landslide pixels in the actual WRF forecast models. The distribution of these distances reveals a strong tendency for new probable areas to occur close or immediately adjacent to existing probable landslide areas, with a median distance of 5 m (corresponding to adjacent pixels due to 5 m grid resolution).

## Discussion

Our study advances landslide hazard assessment by quantifying how dynamic rainfall patterns modulate both landslide susceptibility and intensity, moving beyond traditional static models. Topographic and land cover factors like slope and vegetation type primarily determine the spatial extent of landslide locations, which aligns with previous research in Aotearoa New Zealand by Smith et al.^9^, showing that slope and land cover had the largest influence on landslide susceptibility given a sufficient trigger. On the other hand, rainfall dynamics—especially maximum 24-h totals—govern the intensity of landsliding. This distinction is critical: building upon previous innovations in integrated space–time approaches^10,57,58^, advancing beyond binary susceptibility modelling is an important step towards a more holistic landslide hazard assessment. By jointly modelling both landslide occurrence and area with static geo-environmental factors and dynamic rainfall covariates, we directly capture spatial, temporal, and intensity dimensions of landslide hazards. Previous studies in Aotearoa New Zealand have demonstrated that landslide numbers increase with rainfall intensity, particularly on pasture^43,59,60^. However, limited quantitative evidence directly links rainfall patterns to landslide intensity. Our models reveal a clear threshold: landslide probability and intensity increase linearly with rainfall up to ~ 300 mm/24 h, after which they plateau (Fig. 3). Once this threshold is exceeded, additional rainfall contributes minimally to increased susceptibility. This observation is consistent with recent analysis of Cyclone Gabrielle triggered landslides by Massey et al.^56^, who found that 24-h triggering rainfall becomes less important relative to other susceptibility factors at intensities exceeding 220 mm. Total landslide area follows a comparable nonlinear pattern, with linear scaling ceasing beyond 300 mm/24 h. This non-linear behaviour is crucial for forecasting; a 26–32% increase in areas receiving extreme rainfall (> 95th percentile) translates into a disproportionate 14–26% increase in high-susceptibility (> 95th percentile) landslide zones and 14–34% increase in extreme-density (> 95th percentile) regions. Over the whole study area, this translates to an overall increase of total landslide numbers of ~ 90,000 for the deterministic and ~ 50,000 for the ensemble mean design storms. For the Hawke’s Bay and Tairāwhiti regions, our model estimates ~ 684,000 landslides under the actual Gabrielle rainfall. Our prediction therefore aligns with the North Island-wide estimate of > 800,000 landslides from Massey et al.^56^, and highlights that our study area was most severely impacted by Cyclone Gabrielle.

Previous case studies conducted in the Hawke’s Bay region^61,62^ demonstrated the critical role of antecedent soil moisture in lowering failure thresholds. However, our study revealed a non-monotonic relationship between pre-event rainfall and landslide probability, contrasting the conventional understanding that wetter antecedent conditions increase landslide susceptibility^6,7^. Beyond ~ 150 mm of 35-day cumulative rainfall, landslide likelihood decreased (Fig. S3). We suggest this could be due to: (1) Cyclone Hale, which impacted the Tairāwhiti Region 33 days prior to Cyclone Gabrielle, triggering slope failures that depleted the available regolith and increased terrain resistance^63^; and/or (2) persistent high-rainfall zones may correlate with stabilising factors like dense vegetation or resistant geology shaped by long-term climate adaptation. Such climate-landscape-vegetation feedbacks may not be fully captured by our statistical modelling approach.

Cyclone Gabrielle is one of the most extreme rainfall events recorded in the Hawke’s Bay and Tairāwhiti regions and places it alongside the most severe historical storms like Cyclone Bola in 1988. Cyclone Gabrielle produced 24-h rainfall accumulations up to twice as high as the previous major event in isolated areas of Hawke’s Bay^64^. As argued by Stone et al.^26^, Cyclone Gabrielle is an useful ‘design event’ for hazard assessment and can be considered among the most severe class storms imaginable under current climate conditions. Cyclone Gabrielle is therefore particularly well suited for future storylines and enables valuable projections of future landslide hazards and quantification of their intensification in a warming climate.

The storyline approach offers several strengths for assessing landslide hazards under climate change. By framing Cyclone Gabrielle as a future design storm, it can improve risk awareness by demonstrating that a Gabrielle-like event under + 2 °C warming could trigger up to 90,000 additional landslides, making the scale of future hazards tangible. Crucially, our results show that new landslide-prone areas tend to cluster near existing ones, strengthening decision-making by highlighting that climate change will likely compound risks in already vulnerable locations rather than create entirely new landslide hazard zones.

Importantly, the model response due to increasing rainfall amounts is strongly dependent on the local topography and environmental conditions (see Supplementary Figs. S4, S5). Vulnerable slopes between 20 to 40 degrees experience a disproportionate increase in both susceptibility and intensity compared to flatter slopes while higher forest proportion in a slope unit (both exotic and indigenous) greatly reduced the model response to increasing rainfall (Supplementary Figs. S4, S5). Targeted silvopastoralism on the identified vulnerable slopes represents a proven effective measure to reduce landslide susceptibility^65^ and could be a potential mitigation strategy to counteract projected increases in landslide susceptibility and intensity in already vulnerable locations. This supports prioritising adaptation in known hotspots, where intensified landslide densities present significant management challenges beyond site-specific assessments. Beyond the loss of productive land, coalescing landslide clusters threaten downstream infrastructure and human safety, while elevated sediment delivery exacerbates flood severity.

Research examining the link between climate change and landslides in Aotearoa New Zealand remains limited, with only two studies specifically addressing the Hawke’s Bay and Tairāwhiti regions using downscaled climate projection data. Schmidt and Glade^66^ linked downscaled GCM outputs to regional landslide threshold models for the Wellington and Hawke’s Bay region, predicting a trend toward decreased rainfall-triggered landslide activity by the end of the century due to decreased winter season precipitation. Neverman et al.^67^ developed a national-scale modelling framework that explicitly accounts for mass movement erosion processes, projecting substantial increases in suspended sediment loads primarily driven by shallow landsliding by the of the end-century for Hawke’s Bay and Tairāwhiti. These contrasting findings highlight the need for continued research to better constrain climate change impacts on landslide hazards in this region. Our event-based analysis supports the findings of Neverman et al.^67^, suggesting that the thermodynamic intensification of future extreme rainfall events will likely drive an overall increase in landslide susceptibility and intensity.

Our storyline approach provides a way to partition uncertainty by focusing on the thermodynamic signal of a warmer, moister atmosphere rather than the more uncertain changes in storm dynamics^26^. Recent high-resolution projections for Aotearoa New Zealand^68^ indicate that while cyclone frequency may not change robustly, tropical cyclone intensity in the southwest Pacific is expected to increase by 30–35% by the end of the century, underscoring the plausibility of amplified cyclone related rainfall-driven landslide hazards. Finally, using high-resolution modelling outputs of a single extreme event specifically designed for Aotearoa New Zealand^26^ provides realistic and plausible storylines of a future Gabrielle-like event. Nevertheless, limitations and uncertainties remain. Our statistical landslide model is conditioned on a single extreme event, which restricts generalisation across diverse storm types and characteristics. While our event-specific landslide model performed well, multi-temporal inventories are needed to robustly represent storm variability and to capture the role of antecedent conditions. Recent studies^10,57^demonstrated that multi-temporal inventories can be integrated within spatio-temporal GAM frameworks by treating successive landslide events as repeated observations tied to time-varying rainfall covariates. Such frameworks allow for rigorous cross-validation across both space and time, while effectively disentangling the distinct impacts of antecedent and triggering rainfall characteristics. The impact of antecedent rainfall—complicated here by the earlier impacts of Cyclone Hale—requires deeper investigation, particularly regarding the manner in which antecedent moisture modulates landslide activity during extreme rainfall. This is critical as previous modelling results^27,69^ suggest that reduced antecedent moisture conditions could counterbalance the increased landslide probabilities caused by intensified heavier triggering rainfall to some extent. Further, while the log-gaussian intensity model is straightforward to implement and the mean landslide size is well represented, the extreme tails of the landslide size frequency might be underestimated. Our storyline approach focuses on the thermodynamic amplification (+ 2 °C warming) of ex-tropical cyclone Gabrielle impacting Aotearoa New Zealand, while assuming unchanged storm dynamics. Future research should therefore extend this to consider changes in storm dynamics, alternative warming pathways and include other types of weather systems (e.g. atmospheric rivers, frontal systems, convective storms) resulting in extreme rainfall over Aotearoa New Zealand. However, capturing future changes in fine-scale precipitation extremes remains challenging in Aotearoa New Zealand, as Rampal et al.^33^ argue such changes are inherently unpredictable.

Despite these limitations, this study makes important contributions to landslide hazard assessment under climate change. By leveraging a comprehensive event-based inventory and high-resolution rainfall data we quantify both susceptibility and intensity of landsliding under present and future storm conditions. The application of interpretable GAMs enabled the discovery of complex, non-linear relationships and allows us to interrogate the model response of different interacting covariates. Crucially, focusing on a single extreme event enables the use of high-resolution simulations and allows us to realistically represent the geomorphic imprint of a major storm system. The event-based storyline approach provides an effective way to explore plausible futures and communicate related hazards in tangible terms. Our findings point to significant increases in landslide hazards under warming, likely concentrated in existing hotspots. Given the high probability of another Gabrielle-like storm in the coming decades (80% probability in the next 50 years^70^), the outputs from this research are of immediate practical importance. Our models quantify the degree of expected change and highlight the specific areas most susceptible to landslide intensification under a warmer climate. These findings allow adaptation efforts to be targeted effectively, providing an essential evidence base to inform land-use planning, prioritise mitigation investment, and enhance resilience in Aotearoa New Zealand.

## Supplementary Information

Supplementary Information.

## Data Availability

The generated data for this study is available from the corresponding author.
