# Supplementary material for: Increasing landslide susceptibility and intensity under climate change for Aotearoa New Zealand
Source: Sci Rep. 2026 Apr 7;16:11683. doi: 10.1038/s41598-026-46684-7 (PMC13062160; doi:10.1038/s41598-026-46684-7)
Supplement: Supplementary file 1 — Supplementary Information. [file 41598_2026_46684_MOESM1_ESM.docx]

**Supporting information**

# Overview of methodological workflow

The methodological workflow (Fig. S1) consists of data preparation, statistical modelling to quantify landslide susceptibility and intensity under Cyclone Gabrielle and future +2 °C design storms predictions. Following quality control, a subset of mapped landslides from Hawke’s Bay and Tairāwhiti was combined with high-resolution rainfall and static geomorphic variables. Two mapping units—grid cells and slope units—were employed to capture both fine-scale and geomorphologically relevant terrain characteristics. Generalised Additive Models (GAMs) were developed for binary susceptibility (grid- and slope unit based) and landslide intensity (slope unit based). The model's performance was assessed through both random and spatial cross-validation. The calibrated models were then driven with Weather Research and Forecasting (WRF) simulations of Cyclone Gabrielle and +2 °C design storms to generate landslide susceptibility, intensity, and density predictions. Additional details of the relevant workflow steps are presented in the following sections.


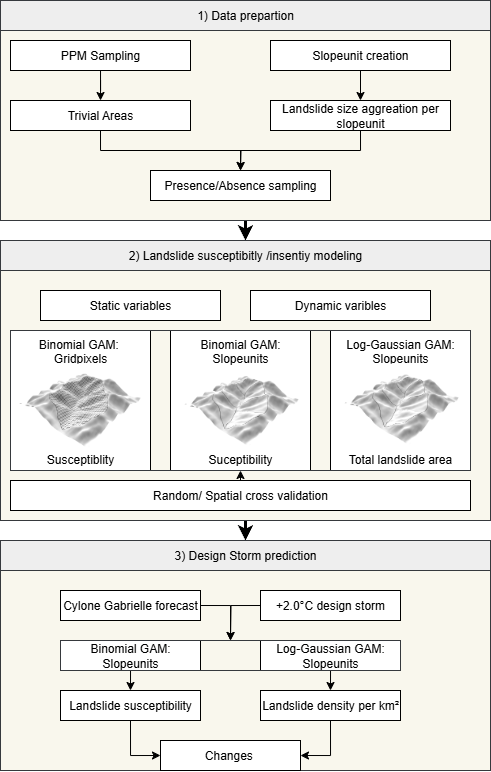


Figure S1: Overview of methodological workflow

# Data preparation and Pre-processing

From the 663 grid cells mapped following the event, we selected a subset of 182 quality-controlled grid cells from the Cyclone Gabrielle dataset within Hawkes Bay and Tairāwhiti regions. We further narrowed down the initial landslide points selecting only landslides mapped as ‘flow’ and ‘slide’ movement type. As a result, we obtain 43,336 landslide points. For the grid pixel model we employ probability-proportional-to-size (PPS) sampling as proposed by Steger et al.^1^. As the actual surface area of steep terrain is underrepresented in a 2D planar view this approach allows us to proportionally generate samples based on true surface area of the terrain. Further we mask out flat terrain (slope <3.5° ) as trivial areas ^2^ to sample a random balanced set of non-landslide points within the mapped grid cells. Covariates were extracted at landslide centroid locations

For the slope unit models, continuous topographic covariates were aggregated by the mean and standard deviation. We aggregated the categorical variable rock type via majority classification. Land use variables showed more distinct variability within slope units, hence we sampled the land-use variables as continuous variables, representing the percentage area of the given land use class per slope unit. We categorised land use into three dominant classes Harvested Forest, Indigenous Forest and Exotic Forest. Pasture serves as the baseline land use class, implicitly representing 100% coverage when all three forest classes exhibit 0% occurrence. This produced a balanced presence/absence slope unit dataset inside the mapped grid cells (16,620 slope units each).

# Slope unit derivation

Grid cells are computationally efficient and straightforward to implement due to their uniform grid structure, however, their artificial geometry often fails to conform to natural geomorphological boundaries, leading to oversimplification of terrain heterogeneity and reduced spatial accuracy. Conversely, slope units—delineated by hydrological boundaries (e.g. ridges, valleys)—better capture geomorphic processes and slope stability mechanisms, enhancing geomorphological relevance and predictive accuracy in susceptibility models. Yet, their delineation requires computationally intensive workflows and their polygonal homogeneity may overlook intra-unit heterogeneity^3^. To integrate strengths of each approach we develop separate grid-pixel and slope-unit models for susceptibility and used the slope-units for the landslide intensity model. We used the r.slopeunit algorithm by Alvioli et al.^4^ to divide our study area into 437,039 slope units, which have a mean size of ~0.051 km² and a standard deviation of ~0.039 km². The following parameters were used for the r.slopeunits algorithm: circular variance=0.2, minimum area=20’000 m².

# Variable selection and assessment of variable importance

Starting with static covariates as a baseline, we iteratively incorporated dynamic rainfall covariates from short-term preparatory, and long-term preparatory groups (see Table S1). For the short-term antecedent rainfall, we considered 7-, 15-, 25- and 35-day time windows. The most relevant covariates for each group were selected based on the lowest Akaike Information Criterion^5^ (AIC) across all three models. Further we make use of the automatic term selection implemented in the mgcv package using a double penalty approach ^6^. Covariates which effectively shrunk to zero were removed from the fitted model.

Following Goetz et al.^7^ and Knevels et al.^8^, we use the decrease in deviance explained to assess the variable importance of each covariate. Therefore, we evaluated the relative importance of individual predictors by comparing the deviance explained by a complete model to the reduced model. Variables whose exclusion resulted in a pronounced reduction in explained deviance, can be interpreted as having a greater relative influence on the model goodness of fit.

Table S1: Summary table of static and dynamic model predictors and final predictor selection for each GAM model. Covariates are represented as their mean (μ), standard deviation (σ), majority or their proportion in each in slope unit.

| Considered Predictor | Binary:Gridpixel | Binary:Slopeunit | Log-gaussian:Slopeunit |
| --- | --- | --- | --- |
| Slope | ✔ | ✔ (μ, σ) | ✔ (μ, σ) |
| Aspect | ✔ | ✔ (μ, σ) | ✔ (μ, σ) |
| Convergence Index | ✔ | **X** | **X** |
| Landuse | ✔(categorical) | **X** | **X** |
| Rocktype | ✔(categorical) | ✔ (majority) | ✔ (majority) |
| Exotic Forest | **X** | ✔(proportion) | ✔(proportion) |
| Indigenous Forest | **X** | ✔(proportion) | ✔(proportion) |
| Harvested Forest | **X** | ✔(proportion) | **X** |
| 24 hour max. rainfall | ✔ | ✔ | ✔ |
| Pre-event rainfall | ✔ (35 day) | ✔ (35 day) | ✔ (35 day) |
| Heavy rainfall frequency | **X** | **X** | **X** |
| Heavy rainfall | **X** | **X** | **X** |
| Annual rainfall | **X** | **X** | **X** |
| Slopeunit area | **X** | ✔* | ✔ |

*The slopeunit area for the binary slopeunit models was only used for model fitting and zeroed out for model predictions

The analysis of the variable importance for both landslide susceptibility and intensity models are presented in Figure S2.


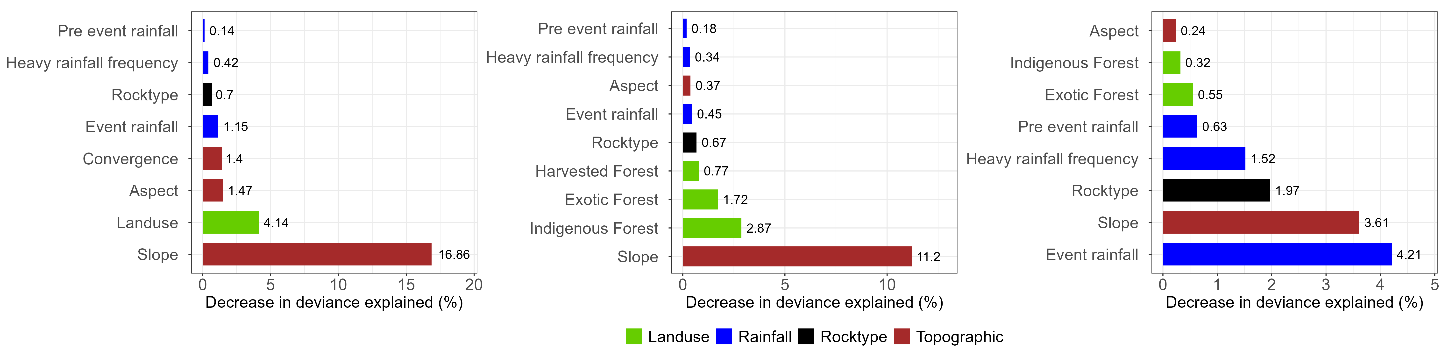


Figure S2: Importance of variables shown by the decrease in deviance explained for a) binary grid pixel, b) binary slope unit and c) log gaussian models.

# Rainfall-landslide relationships

Partial dependence plots show key insights into rainfall-landslide relationships (Fig. S3).


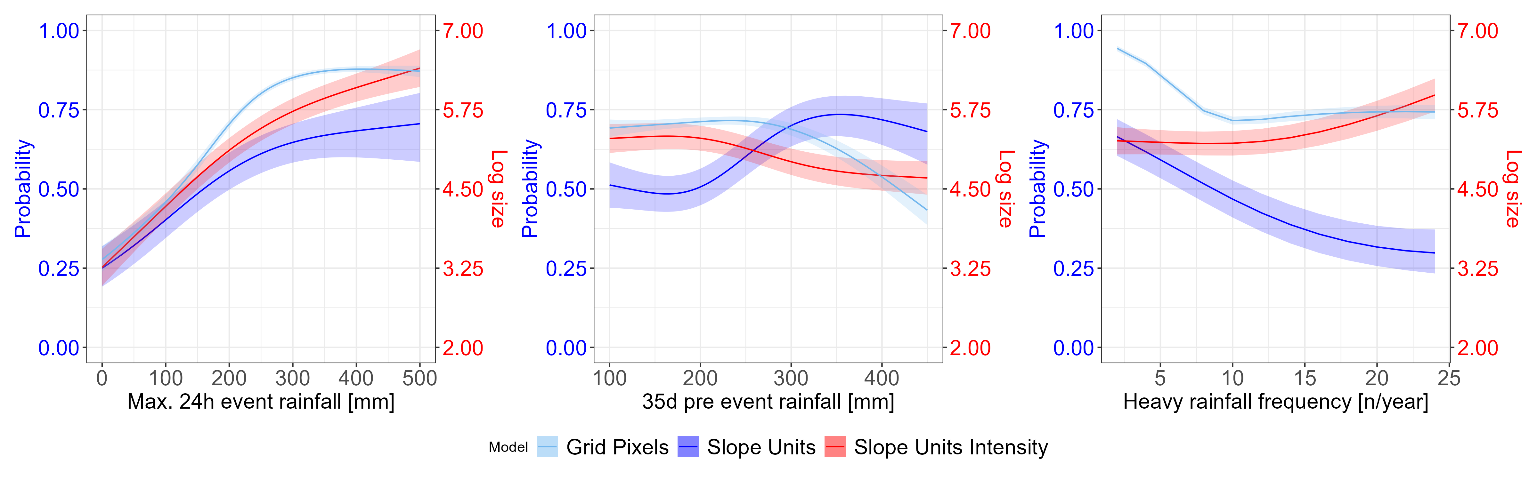


Figure S3 : Partial dependence plots for rainfall variables showing the relationship of the selected variable and model response, while holding the remaining variables at their average. For the binary gridpixel and slopeunit models the model outcome is expressed at the response scale (probability), for the log gaussian slopeunit model the model results is represented as the planimetric landslide area per slope unit on the log scale.

We used 2D contour plots of the partial effects (Figure S4S4, S5 ) to visualise the combined effects of the triggering rainfall variables and the remaining continuous environmental variables on the model outcome. Notably, we observe strong variations in the model outcome for the different variables for the same triggering rainfall amount. For example, combinations of extreme rainfall (>300 mm/24h) and steep slopes (>20°) yielded the highest landslide probabilities (>0.9) in the binary slope-unit model, while flat areas exhibited near-zero probabilities. Aspect exerted much lower influence compared to slope steepness.


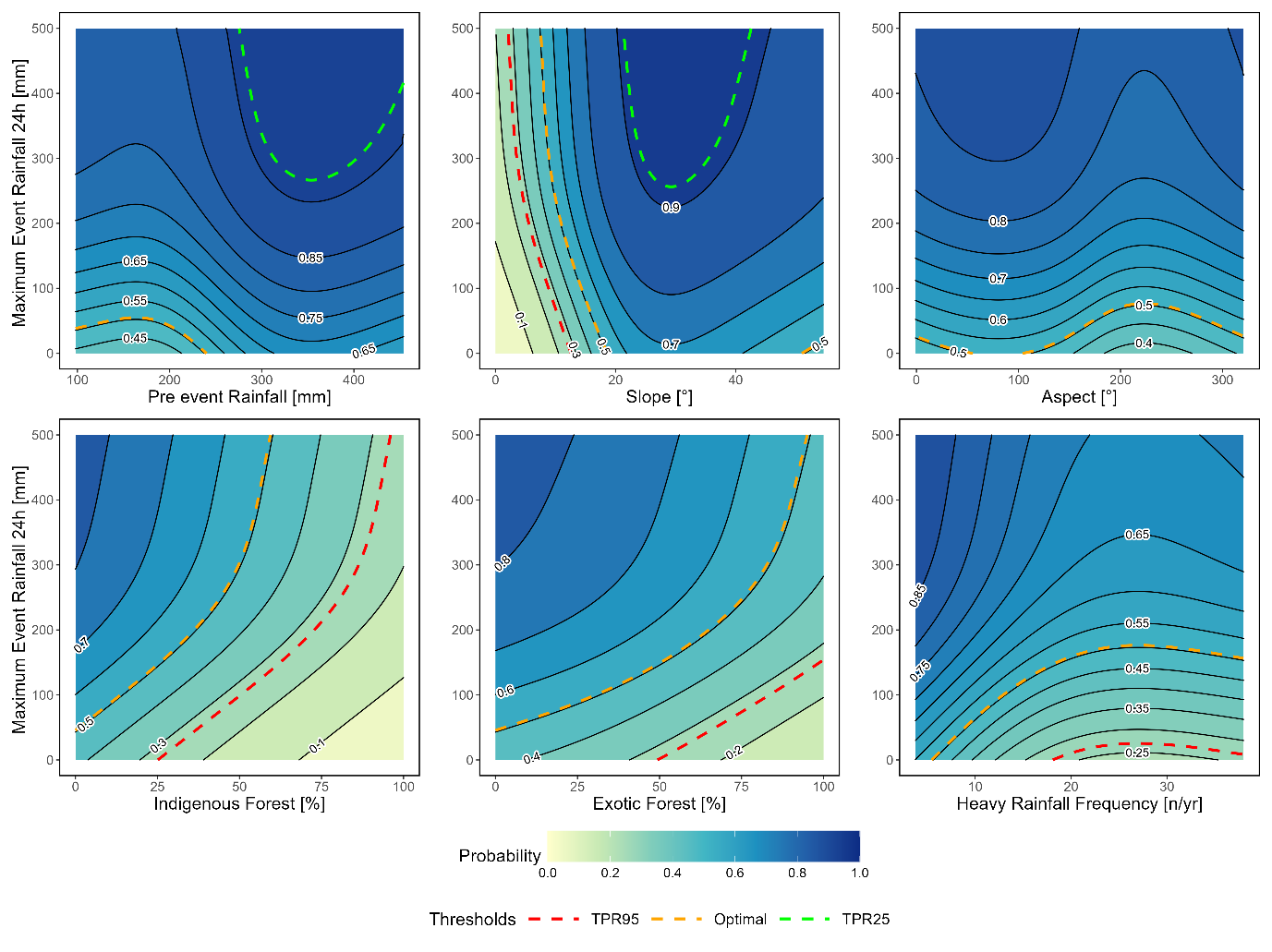


Figure S4: 2D Partial dependence plots for the binary slopeunit model showing the relationship of max 24h. event rainfall and selected variables on the model outcome while holding the remaining variables at their average. The corresponding thresholds (TPR95, Optimal, TPR25) are marked with dashed lines, b) the outcome of the log-gaussian slopeunit model


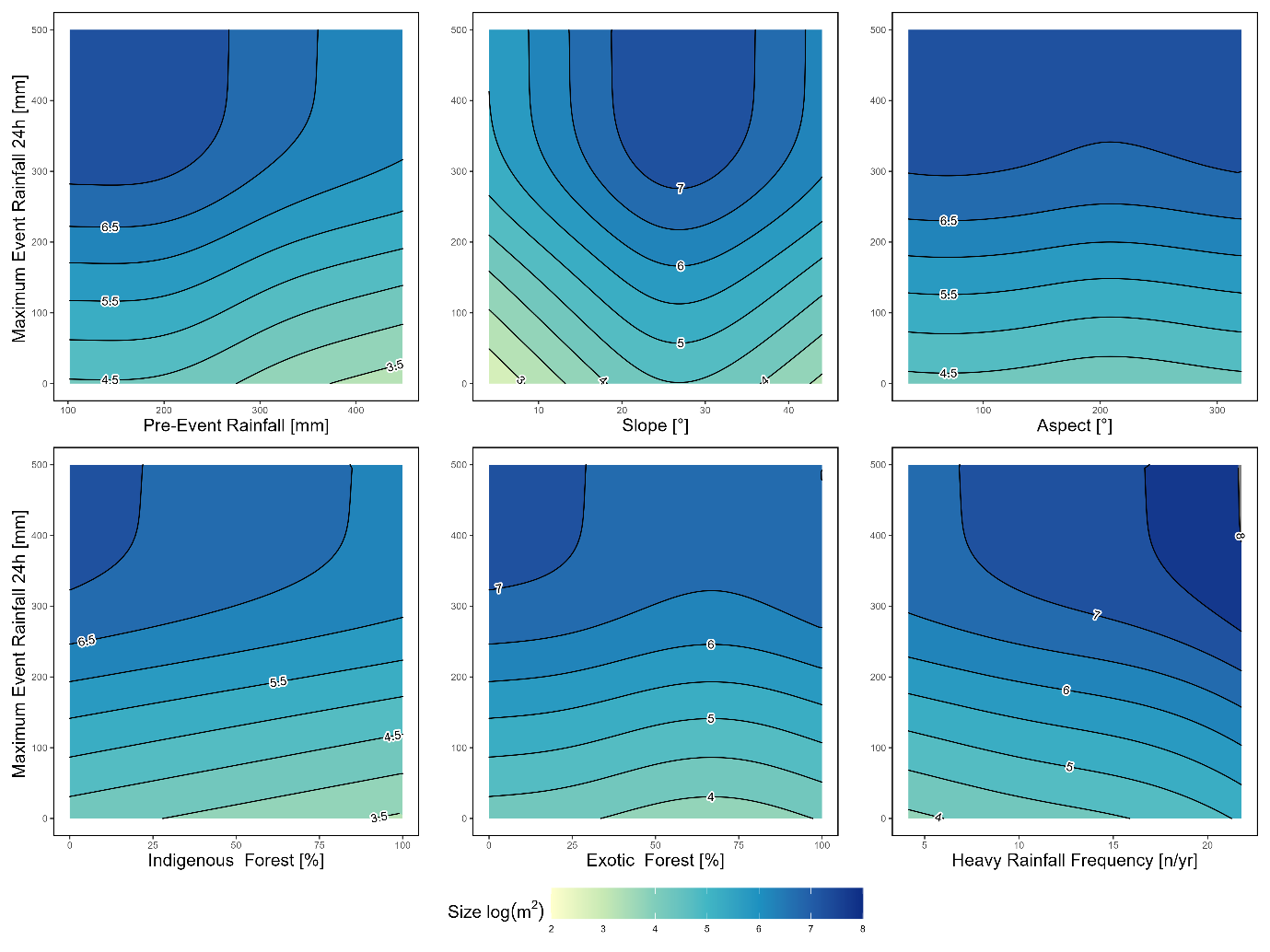


Figure S5: 2D Partial dependence plots for the log-gaussian slopeunit model showing the relationship of max 24h. event rainfall and selected variables on the model outcome while holding the remaining variables at their average.

# Thresholding

We employ three quantitative thresholds derived from Receiver Operating Characteristic (ROC) analysis as proposed by Steger et al.^10^ to visualise the landslide predictions spatially (Fig. S6). These thresholds are based on true positive rates (TPR) and false positive rates (FPR): (1) TPR95 prioritises capturing nearly all landslides but with higher false alarms; (2) OPT, determined via the Youden index^11^, optimises the trade-off between TPR and FPR, (3) TPR25 minimises false alarms rates (FAR) at the cost of reduced landslide detection. These thresholds are applied to probability-based predictions, generating classified maps that highlight areas exceeding critical landslide-triggering conditions. The spatialised thresholds enable intuitive interpretation of susceptibility levels.

Quantitative thresholds based on the AUC curve were defined to categorize susceptibility levels and allow us to spatially relate the amount of precipitation required to exceed a certain threshold. In Figure S6 these thresholds are visualised based on true positive and false alarm rates: TPR95 (95.00% true positive rate (TPR), 39.30% false alarm rate (FAR), probability 0.27), Optimal (Youden Index: 84.20% TPR, 22.9.40% FAR, probability 0.51), and TPR25 (25.00% TPR, 1.10% FAR, probability 0.91. Areas exceeding the TPR25 threshold experienced significantly higher rainfall (median of approximately 280 mm/24h) compared to areas below the TPR95 threshold (140 mm/24h). The spatial patterns of the different thresholds visualised in the zoomed in area of Fig.S6 show the influence of the local terrain characteristics on the threshold distribution. Exceedance of the highest threshold (TPR 25) is mostly restricted to highly susceptible terrain, particularly steep slopes.


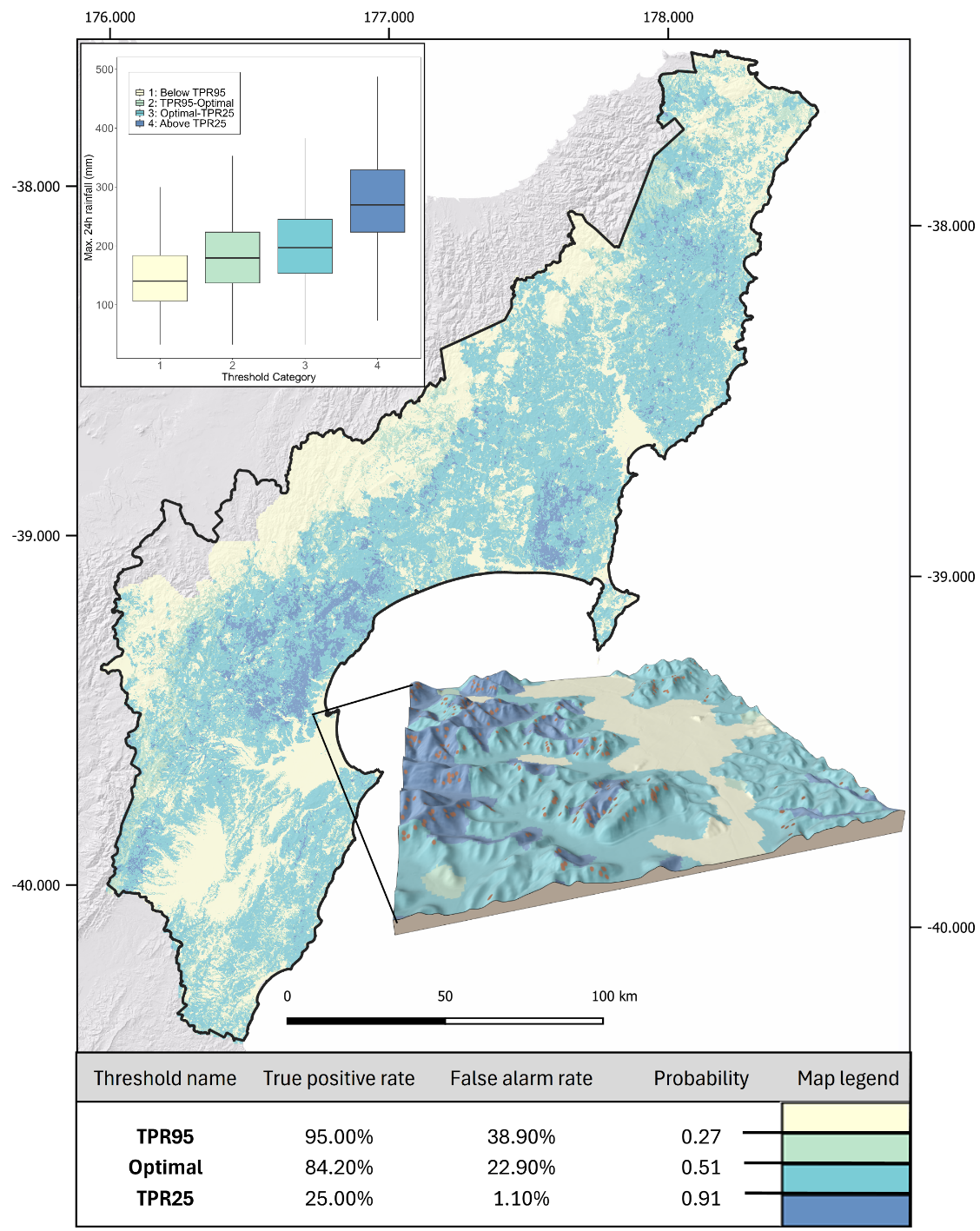


Figure S6: Quantitative thresholds based on the true positive and false negatives rates to visualise thresholds exceedance for Cyclone Gabrielle rainfall. The TPR95 threshold relates to a very high number of true positive values (95% exceed this thresholds) but also includes a high number of false positives. The optimal threshold (Youden index) gives an optimal balance between true positive and false negatives (maximises the sum of specificity and sensitivity). The TPR25 is characterised by a low true positive rate (75 % of all landslides have lower probability) and relates to a high probability score and a very low false alarm rate.

# Landslide storylines for +2.0°C design storms

We use descending cumulative frequency distributions plots (Fig. S7) to show the increase in higher rainfall intensity over the study area under +2.0°C design storms (deterministic and ensemble mean), expanding the spatial extent exceeding susceptibility and density thresholds.


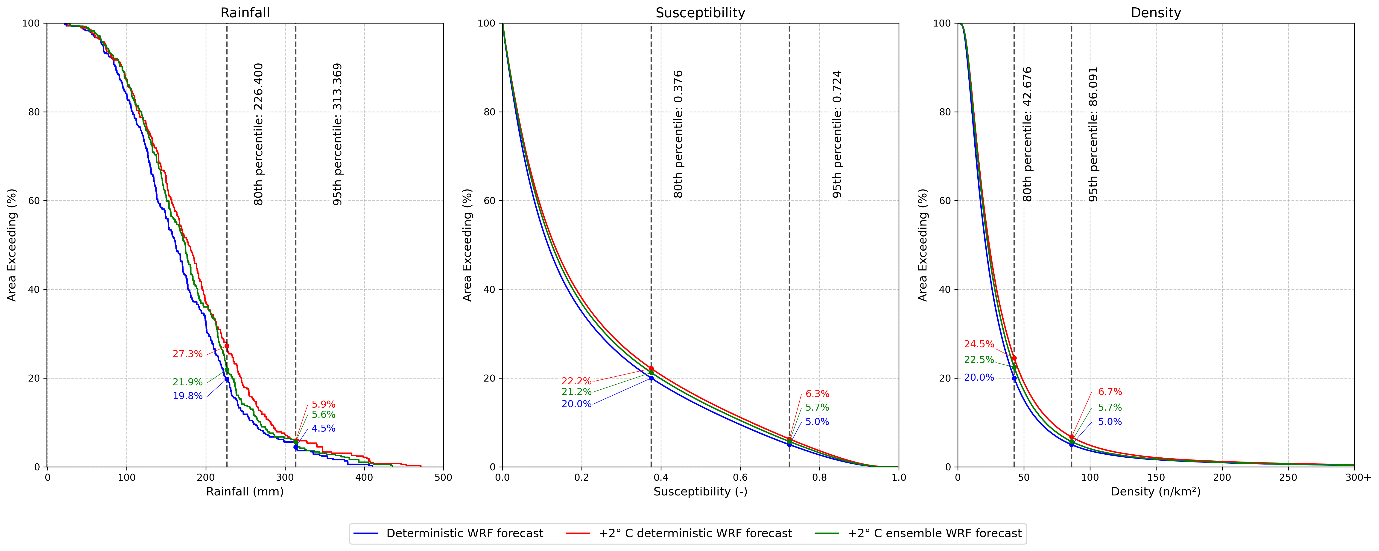


Figure S7: Descending cumulative frequency plots showing the percentage of the study area exceeding values at a given threshold for a) rainfall distribution b) susceptibility and c) landslide densities. Dashed lines locate the threshold at the 95% and 80% percentile for the corresponding target variable.

# References

1. Steger, S. *et al.* Correlation does not imply geomorphic causation in data-driven landslide susceptibility modelling–Benefits of exploring landslide data collection effects. *Sci. Total Environ.* **776**, 145935 (2021).

2. Steger, S. & Glade, T. The Challenge of “Trivial Areas” in Statistical Landslide Susceptibility Modelling. in *Advancing Culture of Living with Landslides* (eds Mikos, M., Tiwari, B., Yin, Y. & Sassa, K.) 803–808 (Springer International Publishing, Cham, 2017). doi:10.1007/978-3-319-53498-5_92.

3. Ba, Q., Chen, Y., Deng, S., Yang, J. & Li, H. A comparison of slope units and grid cells as mapping units for landslide susceptibility assessment. *Earth Sci. Inform.* **11**, 373–388 (2018).

4. Alvioli, M. *et al.* Automatic delineation of geomorphological slope units with r.slopeunits v1.0 and their optimization for landslide susceptibility modeling. *Geosci. Model Dev.* **9**, 3975–3991 (2016).

5. Akaike, H. A new look at the statistical model identification. *IEEE Trans. Autom. Control* **19**, 716–723 (2003).

6. Marra, G. & Wood, S. N. Practical variable selection for generalized additive models. *Comput. Stat. Data Anal.* **55**, 2372–2387 (2011).

7. Goetz, J., Brenning, A., Marcer, M. & Bodin, X. Modeling the precision of structure-from-motion multi-view stereo digital elevation models from repeated close-range aerial surveys. *Remote Sens. Environ.* **210**, 208–216 (2018).

8. Knevels, R. *et al.* Event-based landslide modeling in the Styrian Basin, Austria: accounting for time-varying rainfall and land cover. *Geosciences* **10**, 217 (2020).

9. Brenning, A. Improved spatial analysis and prediction of landslide susceptibility: Practical recommendations. *Landslides Eng. Slopes Prot. Soc. Improv. Underst. Ed. Eberhardt E Froese C Turn. AK Leroueil Taylor Francis Banff Alta. Can.* 789–795 (2012).

10. Steger, S. *et al.* Adopting the margin of stability for space–time landslide prediction–A data-driven approach for generating spatial dynamic thresholds. *Geosci. Front.* **15**, 101822 (2024).

11. Youden, W. J. Index for rating diagnostic tests. *Cancer* **3**, 32–35 (1950).
